# The crucial relationship between miRNA-27 and CSE/H_2_S, and the mechanism of action of GLP-1 in myocardial hypertrophy

**DOI:** 10.7150/ijms.93720

**Published:** 2024-03-31

**Authors:** Shan Gao, Ying Li, Mei-ming Liu, Xue Xiong, Chang-peng Cui, Qing-ji Huo, Ke-xin Li, Xun Sun, Rong Zhang, Di Wu, Bai-yan Li

**Affiliations:** 1 State Key Laboratory of Frigid Zone Cardiovascular Diseases (SKLFZCD), Department of Pharmacology (State Key Laboratory-Province Key Laboratories of Biomedicine-Pharmaceutics of China, Key Laboratory of Cardiovascular Research, Ministry of Education), College of Pharmacy, Harbin Medical University, Harbin 150081, China.; 2 Research Unit of Noninfectious Chronic Diseases in Frigid Zone (2019RU070), Chinese Academy of Medical Sciences, Harbin 150081, China.; 3 Department of Pharmacy, The 2nd Affiliated Hospital of Dalian Medical University, Dalian 116023, China.

**Keywords:** microRNA-27a, cardiac hypertrophy, hydrogen sulfide, cystathionine-γ-lyase, glucagon-like peptide-1 agonist

## Abstract

Cardiac hypertrophy is the most prevalent compensatory heart disease that ultimately leads to spontaneous heart failure. Mounting evidence suggests that microRNAs (miRs) and endogenous hydrogen sulfide (H_2_S) play a crucial role in the regulation of cardiac hypertrophy. In this study, we aimed to investigate whether inhibition of miR-27a could protect against cardiac hypertrophy by modulating H_2_S signaling. We established a model of cardiac hypertrophy by obtaining hypertrophic tissue from mice subjected to transverse aortic constriction (TAC) and from cells treated with angiotensin-II. Molecular alterations in the myocardium were quantified using quantitative real time PCR (qRT-PCR), Western blotting, and ELISA. Morphological changes were characterized by hematoxylin and eosin (HE) staining and Masson's trichrome staining. Functional myocardial changes were assessed using echocardiography. Our results demonstrated that miR-27a levels were elevated, while H_2_S levels were reduced in TAC mice and myocardial hypertrophy. Further luciferase and target scan assays confirmed that cystathionine-γ-lyase (CSE) was a direct target of miR-27a and was negatively regulated by it. Notably, enhancement of H_2_S expression in the heart was observed in mice injected with recombinant adeno-associated virus vector 9 (rAAV9)-anti-miR-27a and in cells transfected with a miR-27a inhibitor during cardiac hypertrophy. However, this effect was abolished by co-transfection with CSE siRNA and the miR-27a inhibitor. Conversely, injecting rAAV9-miR-27a yielded opposite results. Interestingly, our findings demonstrated that glucagon-like peptide-1 (GLP-1) agonists could mitigate myocardial damage by down-regulating miR-27a and up-regulating CSE. In summary, our study suggests that inhibition of miR-27a holds therapeutic promise for the treatment of cardiac hypertrophy by increasing H_2_S levels. Furthermore, our findings unveil a novel mechanism of GLP-1 agonists involving the miR-27a/H_2_S pathway in the management of cardiac hypertrophy.

## Introduction

Cardiac hypertrophy is a response of the heart to various stimuli, categorized as physiological or pathological hypertrophy 1. Physiological hypertrophy refers to normal enlargement of the heart facilitated by capillary expansion to meet the demands of body growth or exercise. In contrast, chronic pathological stimuli such as obesity, heart valve stenosis, and hypertension promote hypertrophic responses that lead to myocardial fibrosis and inflammation, ultimately resulting in heart failure and mortality 2-4. Mechanical stimuli like pressure and volume overload lead to concentric and eccentric hypertrophy, respectively 2. Myocardial hypertrophy is characterized by a substantial increase in the size of cardiomyocytes, accompanied by heightened expression of cardiac markers like natriuretic peptides and β-myosin heavy chain (β-MHC), ultimately leading to myocardial remodeling 3. Given the high mortality linked to heart disease from myocardial hypertrophy, it's crucial to investigate mechanisms and pinpoint new therapeutic targets.

MicroRNAs (miRNAs) are small, single-stranded, non-coding RNA molecules containing 21 to 23 nucleotides. They are essential epigenetic regulators that have significantly advanced our understanding of cardiovascular disease pathogenesis, notably myocardial hypertrophy. For example, miR-155/22 have been found to mediate pro-hypertrophic activity by targeting the calcineurin pathway 5-7. Conversely, miR-208/214 inhibit myocardial fibrosis and hypertrophy by offsetting multi-targets involved in cell development pathways and targeting sirtuin 3 (SIRT3) to induce mitochondrial malfunction 8, 9. The miR-27 family is highly conserved across species and expressed significantly in the heart. It is considered an important epigenetic regulator in cardiac diseases, including non-alcoholic fatty liver disease, tumor development, and myocardial fibrosis 10-12. Herein primarily examines the impact of miR-27 on cardiac hypertrophy, acknowledging its significance as a therapeutic target for heart-related conditions.

Hydrogen sulfide (H_2_S) is an endogenous gas, primarily synthesized through the mutual regulation of cystathionine-β-synthase and cystathionine-γ-lyase (CSE), with CSE as the main H_2_S synthase in the heart 13. H_2_S participates in multiple signal pathways and has been shown to be an important endogenous gas transmitter involved in the protection of nervous and cardiovascular systems 14. It autonomously regulates blood pressure through the pressure reflex pathway, protects against myocardial ischemic injury and ventricular remodeling, and slows down the senescence of cardiomyocytes 15-18. Furthermore, studies suggested that H_2_S and hydrogen sulfide pro-drugs like SG-1002 have a protective effect on myocardial hypertrophy 19, 20. In recent years, the relationship between H_2_S and miRNA has been a topic of interest. For instance, it has been reported that miR-30 can regulate CSE and influence H_2_S expression in the cardiovascular system 21. miR-27 is associated with cardiovascular disease and may be a potential target for the treatment of fibrosis-associated heart disease 12. Therefore, our study predicts and provides the first evidence that miR-27a acts as an upstream suppressor of CSE, elucidating the regulatory relationship in cardiac hypertrophy.

Liraglutide and dulaglutide are artificially synthesized glucagon-like peptide-1 (GLP-1) receptor agonists primarily used in the treatment of type II diabetes. They effectively prevent pancreatic islet β-cell function decline, ensuring long-term blood glucose stability and minimizing adverse drug reactions. Besides managing type II diabetes, liraglutide and dulaglutide have been found to reduce the risk of heart disease in diabetic patients 22, 23. Liraglutide also protects against cardiac hypertrophy through PI3K/Akt1 and AMPK signaling 24. Additionally, liraglutide is closely associated with miR-27a. Hence, we hypothesize that liraglutide and dulaglutide may influence myocardial hypertrophy by regulating miR-27a/CSE/H_2_S.

## Materials and Methods

### Animals

The animal protocols used in the experiments were approved by the Institutional Animal Care and Use Committee of Harbin Medical University, in accordance with the recommendations of the Panel on Euthanasia of the American Veterinary Medical Association and the National Institutes of Health Publication "Guide for the Care and Use of Laboratory Animals". Male C57BL/6 mice weighing 20-25 g were purchased from the Experimental Animal Center of Harbin Medical University and housed under standard animal room conditions (temperature 21 ± 1 ºC and humidity 55-60%), with *ad libitum* access to food and water. To establish the pressure-overload mouse model of cardiac hypertrophy, the mice underwent transverse aortic constriction (TAC) and were available for use in experiments at 4 weeks after TAC surgery.

### Pressure-overload cardiac hypertrophy

Transverse aortic constriction (TAC) was performed to create a pressure-overload mouse model of cardiac hypertrophy. Male mice weighing 20-25 g were anesthetized using 2% avertin administered via intraperitoneal injection. The mice were fixed on the operating table in a supine position, and a cannula was connected to a volume circulation rodent ventilator (Ugo Basile SRL 3) after successful endotracheal intubation. The chest was opened to identify the thoracic aorta, and the aortic arch was tied with a 26-gauge blunt needle using 6/0 silk suture. The chest was then closed after removing the needle. The sham group underwent the TAC procedure without aorta ligation. Echocardiography was performed to assess myocardial hypertrophy at 4 weeks after TAC.

### Echocardiographic test

Transthoracic echocardiography was conducted using an ultrasound (Vevo 2100 imaging system, VisualSonics, Toronto, Canada) to evaluate cardiac function parameters. The heart was imaged in a parasternal short-axis view at the level of the papillary muscles to record parameters using M-mode. The left ventricular systolic/diastolic posterior wall (LVPWs/LVPWd, mm) and interventricular septum systolic/diastolic thickness (IVSs/IVSd, mm) were measured, and left ventricular wall thickness was used as an index of cardiac hypertrophy.

### Histological analysis

Heart tissues fixed with paraformaldehyde were embedded in paraffin and sliced into 4-μm sections. The size and morphological changes of the heart were analyzed using hematoxylin/eosin (HE) staining and Masson trichrome staining. The sections were mounted and scanned using a panoramic scanner (Olympus, Japan).

### Delivery of the miRNA modulator and drugs in mice

Recombinant adeno-associated virus vector 9 (rAAV9) was used as the most efficient vector for myocardial transduction among rAAV vectors. For this purpose, rAAV9-anti-miR-27a, rAAV9-miR-27a, or rAAV9-NC was designed (PackGene Biotech, Guangzhou, China), and the sequences were injected through the tail vein at a dose of 1 × 10^11^ vg per animal 3 weeks before TAC. Additionally, 20 mg/kg of S-propargyl-cysteine (SPRC) was injected intraperitoneally on a daily basis for 8 days in different groups prior to TAC surgery 24.

### Primary culture and treatment of neonatal mouse cardiomyocytes

Neonatal mouse cardiomyocytes were obtained from 1 to 3-day-old mice. Following digestion with pancreatin (Beyotime, Shanghai, China) and Type II collagenase (Gibco, Carlsbad, CA, USA), myocytes were isolated by selective adhesion at a 1.5-hour pre-plating interval. The cardiomyocytes were then maintained in DMEM supplemented with 1% penicillin and streptomycin, 10% fetal bovine serum, and 0.1 mmol/L of 5-bromo-2-deoxyuridine in a 37 °C incubator with 5% CO_2_, following the guidelines outlined in the "UKCCCR Guidelines for the Use of Cell Lines in Cancer Research" 25. miR-27a-3p-mimic, miR-27a-3p-inhibitor, CSE siRNA, and their corresponding controls were synthesized by RIOBIO (Guangzhou, China) and transfected using lipofectamine 2000 transfection reagent (Invitrogen, Carlsbad, CA, USA) under serum-free medium conditions. After 12 hours of transfection, a new serum-free medium with or without angiotensin II (100 μmol/L) was used. After 48 hours of culture, the cells were collected for protein/total RNA extraction.

Cardiomyocytes were seeded in twelve-well culture plates and incubated for 48 hours. The cells were then cultured with serum-free DMEM for another 12 hours before treatment. After treatment with angiotensin II for 48 hours and/or liraglutide/dulaglutide (100 nmol/L) for 24 hours, ANP, BNP, and β-MHC genes were detected by quantitative real-time PCR (qRT-PCR).

### Quantitative Real-Time PCR for mRNAs

Total RNA samples were extracted from cultured neonatal mouse ventricular cardiomyocytes (NMVCs) and cardiac tissue using TRIzol reagent (Invitrogen, Carlsbad, CA, USA). The RNA was reverse-transcribed with cDNA reverse transcription reagent kits (FSQ-101, Toyobo, Japan), and qRT-PCR was carried out using an ABI 7500 fast real-time PCR system (Applied Biosystems) with the SYBR Green PCR Master Mix Kit (04913914001, Roche, Switzerland). The primers for qRT-PCR were synthesized by Sangon Biotech (Shanghai, China), and their sequences are listed in Table 1., U6 or GAPDH was used as an internal control.

### Western blot

Protein samples extracted from mouse hearts or NMVCs were used for immunoblotting analysis. The concentration of the proteins was determined using a BCA Protein Assay Kit (Beyotime, Shanghai, China). Equal amounts of protein (80 mg) were loaded on a 10% SDS-Tris glycine gel electrophoresis and then transferred onto nitrocellulose membranes. After blocking with 5% nonfat milk for 2 hours at room temperature, the membranes were incubated overnight with the corresponding primary antibodies at 4 °C, including anti-CSE (1:1,000, Abnova), anti-ANP (1:1,000, GeneTex), anti-BNP (1:1,000, ABclonal), anti-MHC (1:1,000, SANTA), and anti-TUBULIN (1:1,000, ABclonal). After being washed with PBST three times, each for 10 minutes, the membranes were incubated with secondary antibodies (1:8,000, ABclonal) for 55 minutes at room temperature and washed with PBST again. The results were detected and analyzed using an Odyssey system (LI-COR Biosciences, Lincoln, NE, USA).

### Measurement of cell surface area

Cardiomyocytes were fixed with 4% paraformaldehyde for 15 minutes, permeabilized with 0.4% Triton X-100 for 20 minutes, and then blocked with goat serum for 1 hour at 37 °C. The cells were first incubated with anti-Sarcomeric Alpha Actinin (1:200, Sigma, Cat#A7811) at 4 °C overnight and subsequently with Alexa Fluor 594 (Molecular Probes) for 1 hour at room temperature. Then, the cells were incubated with DAPI (C1005, Beyotime, China) for 10 minutes. The preparations were examined under an immunofluorescence microscope.

### Luciferase reporter assay

Reporter vectors containing miRNA-binding sites were synthesized for luciferase reporter assays (PackGene Biotech, Guangzhou, China). The constructs were inserted into the multiple cloning sites downstream of the luciferase gene (Xhol and Not I sites) in the psiCHECK-2 luciferase reporter vector (Promega, USA). For the luciferase assay, 0.1 mg of luciferase reporters containing the 3'UTR was co-transfected with the appropriate controls or miRNA mimics/inhibitors (miR-27a-3p-mimic, miR-27a-3p-inhibitor) and 10 ng of psiCHECK-2 into HEK293 cells using lipofectamine 2000 transfection reagent (11668027, Invitrogen, USA) according to the manufacturer's instructions 26. Luciferase activity was measured at 48 hours after transfection using a dual-luciferase reporter assay kit (Promega, USA).

### Measurement of H_2_S concentration

H_2_S concentration was measured in blood samples and cell supernatants from different experimental groups. The samples were separated and H_2_S was detected using an ELISA Kit (Jianglai Biology) following the manufacturer's instructions.

### Statistical analysis

Excel and GraphPad Prism 9 were employed for statistical analyses and the creation of graphs. Analyses were conducted solely on studies that provided a minimum of four complete observations (n), as indicated in the figure legends. To assess significant differences pre- and post-treatment or between groups, either paired or unpaired Student's t-tests were utilized. Comparisons across multiple groups were performed using one-way ANOVA. Values for each control and test group were "normalized" to the mean value of the control group. The unit of measure for such normalized data was expressed as fold change relative to the control group's mean, and these were accurately labeled in the figure legends. Averaged data were presented as mean ± SD. A P value of less than 0.05 was deemed indicative of a statistically significant difference.

## Results

### miR-27a/b was up-regulated and CSE was down-regulated in TAC mice and Ang II-induced NMVCs

**Figure 1:**
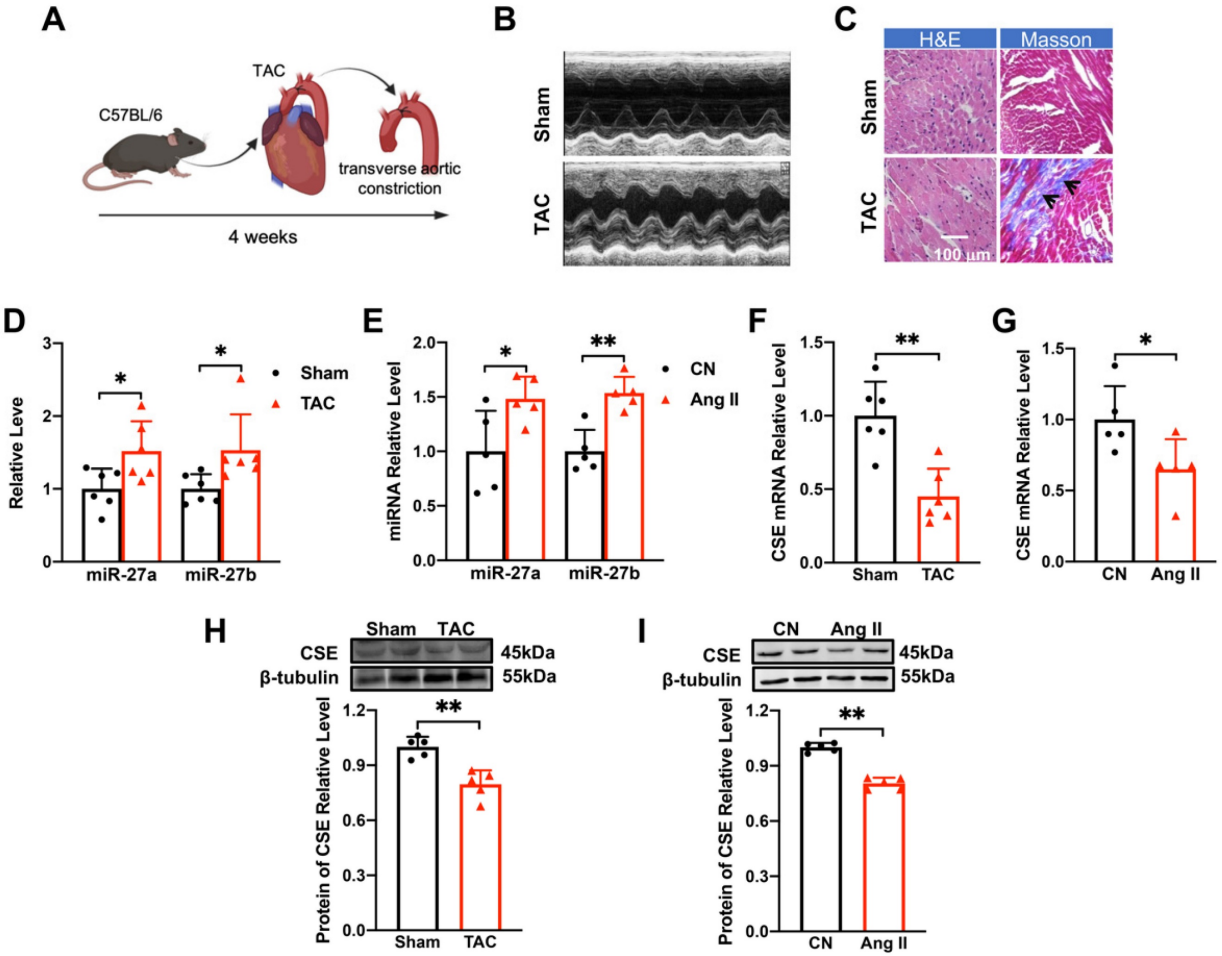
** Changes of miR-27a/b and CSE levels in TAC mice and Ang II-induced NMVCs. (A).** Modeling of myocardial hypertrophy in mice. **(B)** Echocardiograms of mice in the control group (Sham) and TAC group (*n =* 6-10 mice).** (C)** Macroscopic view of Masson and HE-stained transverse sections showing myocardial fibrosis and in control and TAC hearts. HE × 400, Masson × 400. Scale bar: 100 μm (*n =* 3 mice). **(D)** Changes in miR-27a and miR-27b levels in TAC mice compared to the sham group (*n =* 5-6 mice).** (E)** qRT-PCR detecting changes in miR-27a and miR-27b in neonatal mouse ventricular cardiomyocytes (NMVCs) after 48 hours of Ang II treatment (*n =* 5-6 group). **(F-I)** Changes in mRNA and protein levels of CSE in hypertrophy models* in vivo* and *in vitro* (*n =* 5-6 mice or group). Averaged data are presented as mean ± SD; *P < 0.05, **P < 0.01. Note: CN represents the control group, Ang II represents angiotensin II, and TAC represents transverse aortic constriction.

The TAC mouse model is widely used to investigate cardiac hypertrophy (Figure 1A). Echocardiography (Figure 1B) and quantitative analysis of mice at 4 weeks after TAC surgery revealed that the anterior and posterior walls of the left ventricle were significantly thicker during both diastole and systole compared to the sham group (Supplementary Figure 1A-D). Additionally, the heart-body weight ratio (Supplementary Figure 1E) was significantly increased following TAC surgery. Pathological examination of ventricular tissues using Masson and hematoxylin-eosin staining showed collagen deposition (as indicated in the figure) and enlargement of the cross-sectional area, indicating hypertrophic changes (Figure 1C). Moreover, the mRNA levels of hypertrophy-related genes ANP, BNP, and β-MHC, as well as their corresponding protein levels, were up-regulated in response to TAC procedures (Supplementary Figure 1F-H). These findings confirmed the successful establishment of myocardial hypertrophy *in vivo*.

To establish the *in vitro* cellular model of myocardial hypertrophy, NMVCs were exposed to Ang II for 48 hours. This treatment significantly enhanced the hypertrophic indicators at both mRNA and protein levels (Supplementary Figure 1I-L), validating the successful establishment of the *in vitro* model. Furthermore, miR-27a/b expression was significantly up-regulated in both the *in vivo* (Figure 1D) and *in vitro* (Figure 1E) models, suggesting its potential association with myocardial hypertrophy.

CSE, an essential enzyme for H_2_S synthesis in the heart, has been associated with a protective effect against myocardial hypertrophy 17, 18. Surprisingly, the mRNA and protein levels of CSE were decreased in both the *in vivo* and *in vitro* settings of pathological cardiac hypertrophy (Figure 1F-I). In conclusion, our results demonstrate the successful establishment of the TAC mouse model of cardiac hypertrophy and the Ang II-induced *in vitro* cellular model. Additionally, we observed up-regulation of miR-27a/b expression in both models, indicating its potential involvement in myocardial hypertrophy. Interestingly, the down-regulation of CSE suggests a novel role for this enzyme in the development of pathological cardiac hypertrophy.

### CSE is directly targeted by miR-27a/b, exerting a regulatory effect on CSE

**Figure 2:**
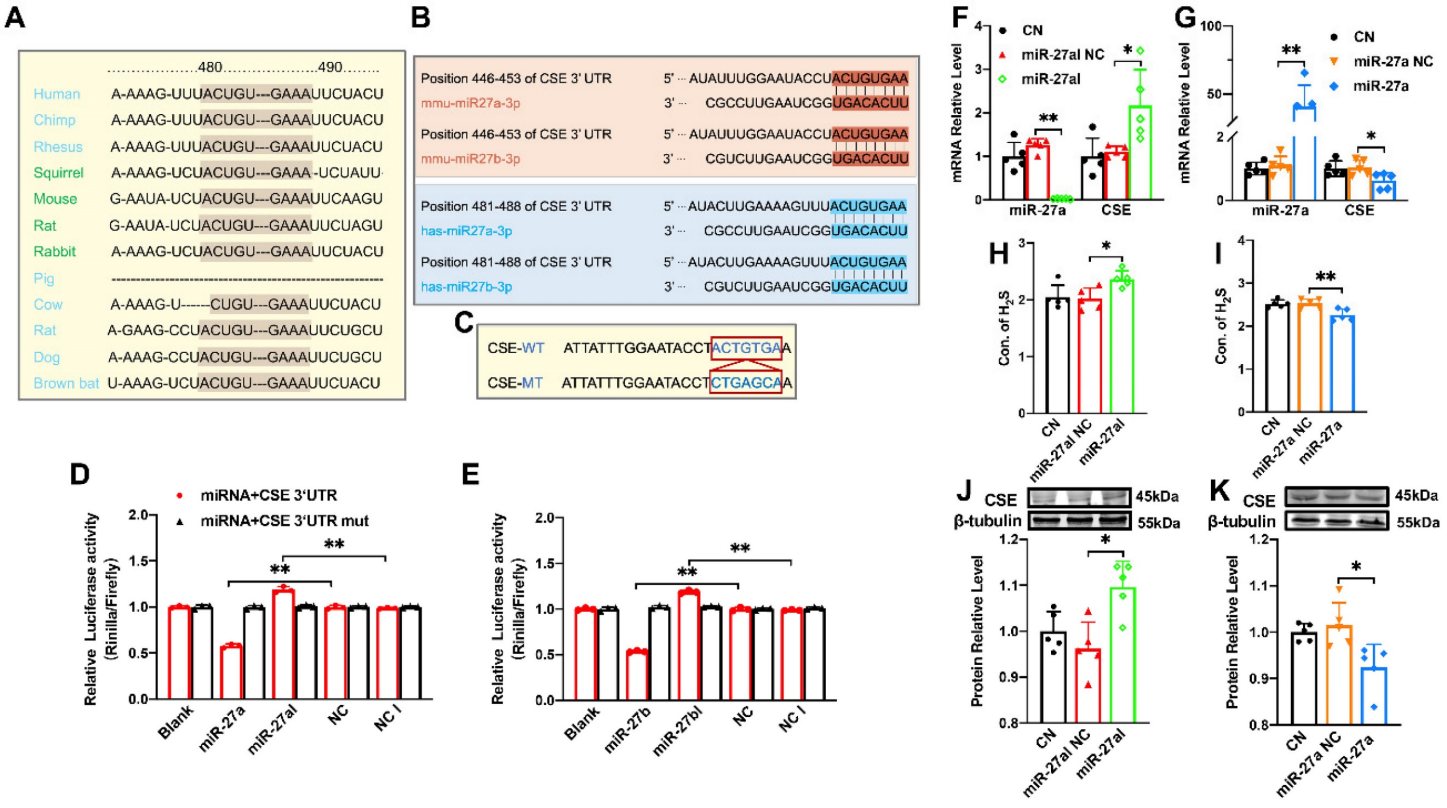
** The regulatory effect of miR-27a on CSE. (A)**. The sequence of miR-27 between 480-490 is highly conserved among humans, rats, mice, rabbits, and other organisms. **(B)**. Sequence alignment between members of the miR-27 family and 3′-UTRs of CSE in humans and mice. The complementary sequences in CSE genes to the miR-27a/b seed regions are highlighted in red and blue, respectively. **(C)**. Constructs of wild-type (WT) and mutant (MT) 3′UTRs of CTH were generated. **(D, E)**. Luciferase reporter gene assay measuring the interactions between miR-27a/b and CSE in HEK293T cells (*n =* 3 group). **(F, G)**. Changes in miR-27a and CSE mRNA expression were determined by qRT-PCR after miR-27a inhibition (miR-27aI) or overexpression (miR-27a) in cells (*n =* 5 group). **(H, I)**. Cell supernatant was collected to assess the alterations in H_2_S expression after silencing or overexpressing miR-27a using the Elisa Kit, compared to the control (CN) and negative control (NC) groups (*n =* 5 group). **(J, K)**. Western blot analysis was carried out to investigate changes in CSE protein expression after miR-27a inhibition or overexpression (*n =* 5 group). The figure legend from **(D)** applies to **(E)**. The data are presented as the mean ± SD; *P < 0.05, **P < 0.01.

The miR-27 gene sequence exhibits high uniformity across various organisms, including humans, mice, and rabbits, with the 480-490 sequence showing consistency (Figure 2A). Additionally, target scan software predicted complementary sequences between miR-27a/b and CSE genes in humans and mice (Figure 2B). To confirm the relationship between miR-27a/b and CSE, we constructed wild-type and mutant CTH (CSE coding gene) (Figure 2C), which were inserted into the dual-luciferase expression plasmid (psiCHECK-2) and co-transfected with miR-27a/b mimic or inhibitor into HEK293 cells. Luciferase results revealed that the fluorescence intensity of wild-type CTH was weakened upon overexpression of miR-27a/b, while the mutant CTH showed the opposite effect (Figure 2D, E). These results suggest that CSE could potentially serve as a target of the miR-27 family.

Since miR-27a and miR-27b share a complementary sequence with CSE, only miR-27a was selected for further experiments. In order to validate the relationship between miR-27a and CSE, myocardial cells were transfected with miR-27a mimic and inhibitor, and the transfection efficiency was confirmed by measuring miR-27a levels. Consistently, transfection with miR-27a mimic resulted in the down-regulation of CSE expression (Figure 2G, K), whereas miR-27a inhibitor transfection showed the opposite effect (Figure 2F, J). Furthermore, the levels of H_2_S in the cell supernatant coincided with the expression of CSE detected by the Elisa Kit (Figure 2H, I). Collectively, these findings suggest an inverse correlation between miR-27a and CSE/H_2_S.

### Silencing miR-27a protects cardiomyocyte hypertrophy

**Figure 3:**
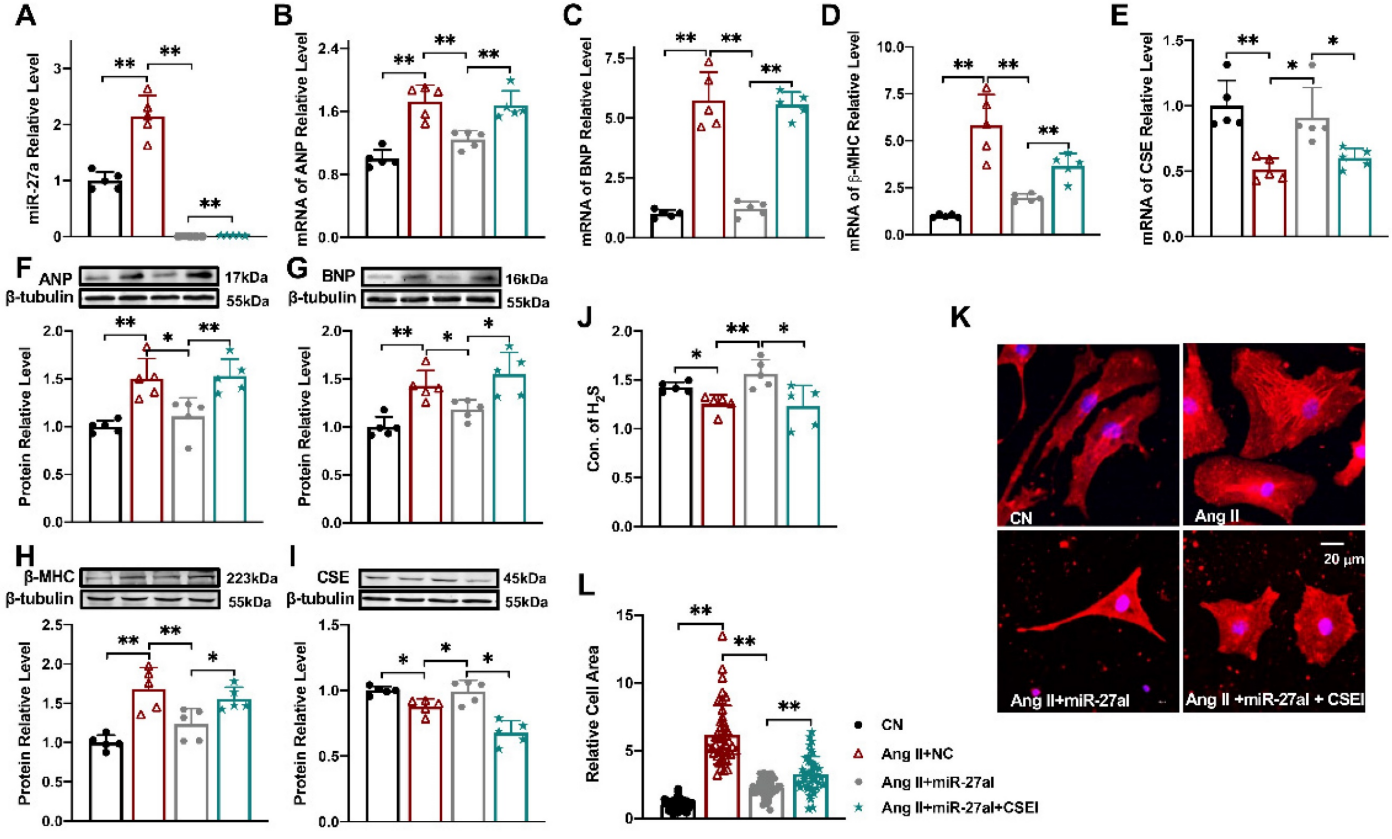
** Silencing miR-27a protects cardiomyocyte hypertrophy.** Relative expression of β-MHC, ANP, BNP, CSE, and miR-27a was assessed in NMVCs using qRT-PCR and Western blot analysis in various experimental conditions: CN, Ang II + negative control (NC), Ang II + miR-27a inhibitor (miR-27aI), and Ang II + miR-27aI + CSE siRNA (CSEI). The mRNA levels **(A-E)** and protein levels **(F-I)** were analyzed (*n =* 5 group). **(J).** H_2_S expression was quantified using an Elisa Kit (*n =* 5-6 group). **(K, L).** Cell area changes were determined by fluorescence imaging and statistical analysis for the four groups: CN, Ang II + NC, Ang II + miR-27aI, and Ang II + miR-27aI + CSEI (*n =* 40-50 cells). The magnification factor used was × 400, with the scale bar representing 20 μm. The figure legend from **(L)** applies to all. The data are expressed as mean ± SD, with statistical significance denoted by *P < 0.05 and **P < 0.01. Notably, CN denotes the control condition, and Ang II refers to angiotensin II.

To investigate the impact of miR-27a on myocardial hypertrophy *in vitro*, we introduced a miR-27a inhibitor into cardiomyocytes to silence miR-27a while establishing the cell hypertrophy model (Figure 3A). Strikingly, miR-27a deficiency mitigated the hypertrophic responses, evident from a reduction in the expression of hypertrophy markers in cardiac tissues, irrespective of mRNA (Figure 3B-D) or protein levels (Figure 3F-H). Additionally, the miR-27a inhibitor also alleviated the suppression of CSE (Figure 3E, I) and H_2_S levels (Figure 3J) induced by Ang II. Notably, morphological observations demonstrated that the miR-27a inhibitor attenuated Ang II-induced pathological cardiac hypertrophy, as evidenced by a decrease in cell surface area (Figure 3K, L). To examine whether the protective effect of miR-27a on myocardial hypertrophy is associated with CSE, we assessed the transfection efficiency of three CSE siRNAs and selected the one with the highest efficiency to silence the expression of CSE in the cells (*P* < 0.01, *n =* 5 group; results not shown). Subsequent co-transfection of the miR-27a inhibitor and CSE siRNA restored the H_2_S level, the expression of hypertrophy markers, and cell surface area in the Ang II treatment group. Taken together, these results suggest that the protective effect of miR-27a inhibition on myocardial hypertrophy is dependent on CSE/H_2_S signaling.

### rAAV9-anti-miR-27a affects myocardial hypertrophy by regulating CSE/H_2_S

**Figure 4:**
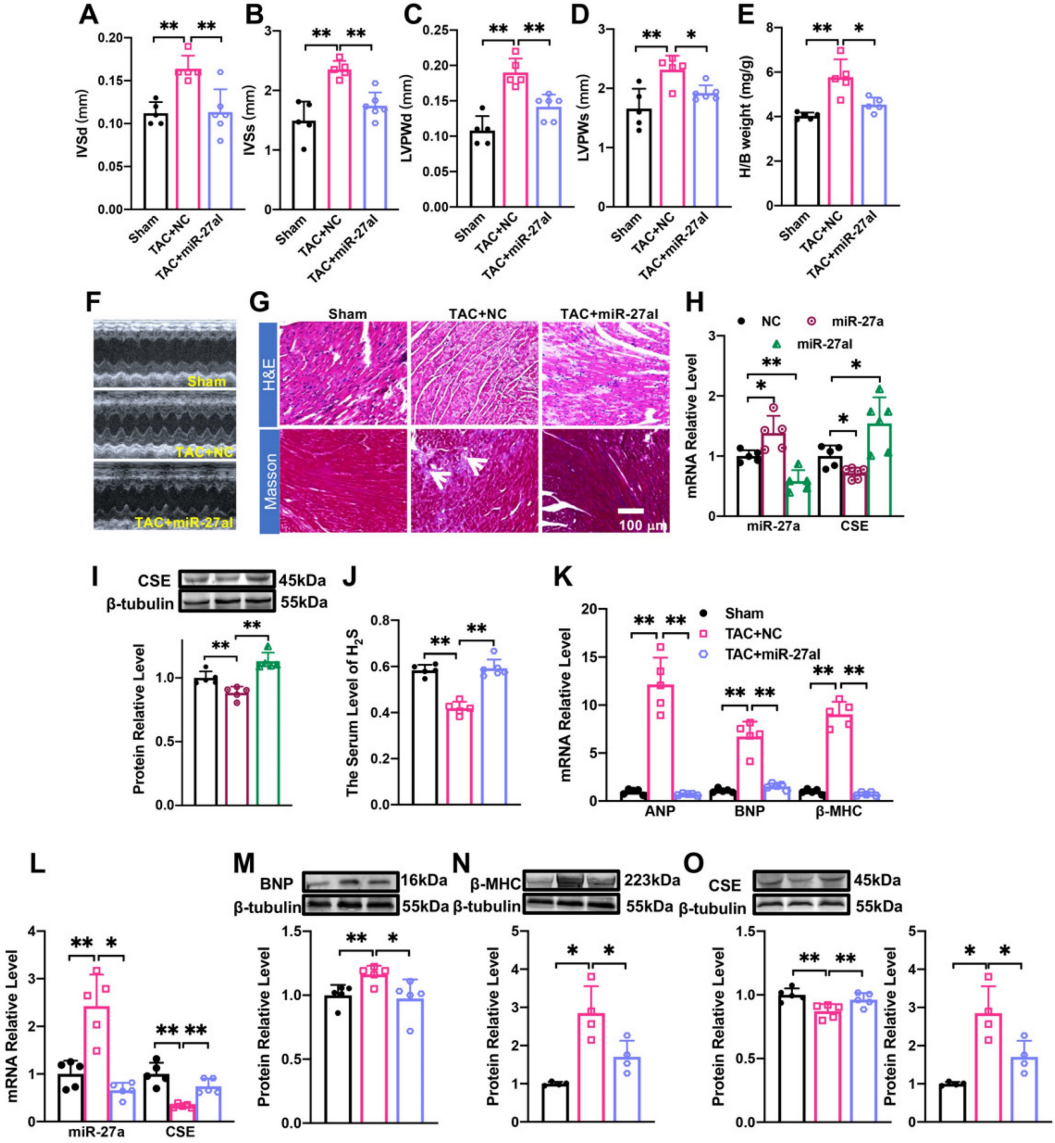
** rAAV9-anti-miR-27a affects myocardial hypertrophy by regulating CSE/H_2_S. (A-E).** Statistical results of interventricular septal thickness in diastole (IVSd, mm) and systole (IVSs, mm), left ventricular posterior wall at end-diastole (LVPWd, mm) and end-systole (LVPWs, mm), Heart/body weight (H/B weight, mg/g) (*n* = 5 mice). **(F).** Echocardiograms of mice in Sham, TAC + NC, TAC+ miR-27aI. **(G).** Masson and HE staining were used to detect transverse sections and myocardial fibrosis in C57BL/6 after injecting rAAV9-anti-miR-27a for three weeks before TAC modeling. HE ×400, Masson ×400. Scale bar represents 100 μm. (*n* = 3 mice). **(H, I).** Detect the mRNA level and protein level of miR-27a and CSE in the heart of C57BL/6 mice after tail vein injection of rAAV9-anti-miR-27a (miR-27aI) and rAAV9-miR-27a (miR-27a) for three weeks. **(K-O).** Four weeks after modeling, qRT-PCR and Western blot experiments were used to detect the mRNA and protein expression changes of hypertrophic factors, CSE and miR-27a compared with Sham and TAC NC group. β-tubulin served as an internal control. (*n* = 4-6 mice). **(J).** Changes in the expression of H_2_S in serum. Figure legend from **(K)** applies to **(J)**, **(L-O)**. Averaged data are presented as the mean ± SD; *P < 0.05, **P < 0.01. Of note, NC negative control, TAC transverse aortic constriction.

We investigated the potential of miR-27a interference in combating cardiac hypertrophy *in vivo*. To specifically inhibit the expression of endogenous miR-27a in the heart, we administered rAAV9-anti-miR-27a (PackGene, Guangzhou, China) through tail vein injection in C57BL/6 mice for 3 weeks. Ultrasound examination was performed on the mice at 4 weeks after TAC surgery to assess changes in heart function. The results revealed that the suppression of miR-27a exacerbated cardiac malfunction, consistent with the* in vitro* findings (Figure 4A-D). Furthermore, it prevented the increase in the heart-body weight ratio following TAC surgery (Figure 4E). These results were further supported by echocardiogram data (Figure 4F). Morphological analysis showed that the cross-sectional area of the heart and myocardial fibrosis (as indicated in the figure) were significantly reduced in the rAAV9-anti-miR-27a group compared to the model group based on Masson and HE staining (Figure 4G). These findings strongly indicated that miR-27a could reverse myocardial hypertrophy *in vivo*.

We then investigated whether the underlying mechanism of miR-27a in myocardial hypertrophy is associated with CSE. To evaluate the specific expression effect of the heart after tail vein injection of adenovirus for 3 weeks, we constructed two viruses: rAAV9-anti-miR-27a and rAAV9-miR-27a (PackGene, Guangzhou, China), both were the preferred vector for cardiac gene interference. qRT-PCR analysis revealed that rAAV9-anti-miR-27a effectively silenced the expression of miR-27a in the heart (Figure 4H) while increasing CSE expression (Figure 4H, I). Conversely, overexpression of miR-27a reversed CSE expression. Four weeks after TAC procedures, mRNA (Figure 4K) and protein (Figure 4M, N) levels of hypertrophy-related genes were significantly lower in mice injected with rAAV9-anti-miR-27a compared to the TAC group. Moreover, the expression of CSE (Figure 4L, O) and H_2_S (Figure 4J) were increased. These findings further confirm that miR-27a inhibition is able to protect against myocardial hypertrophy by up-regulating CSE/H_2_S.

### The effect of miR-27a on myocardial hypertrophy depends on the regulation of CSE/H_2_S

**Figure 5:**
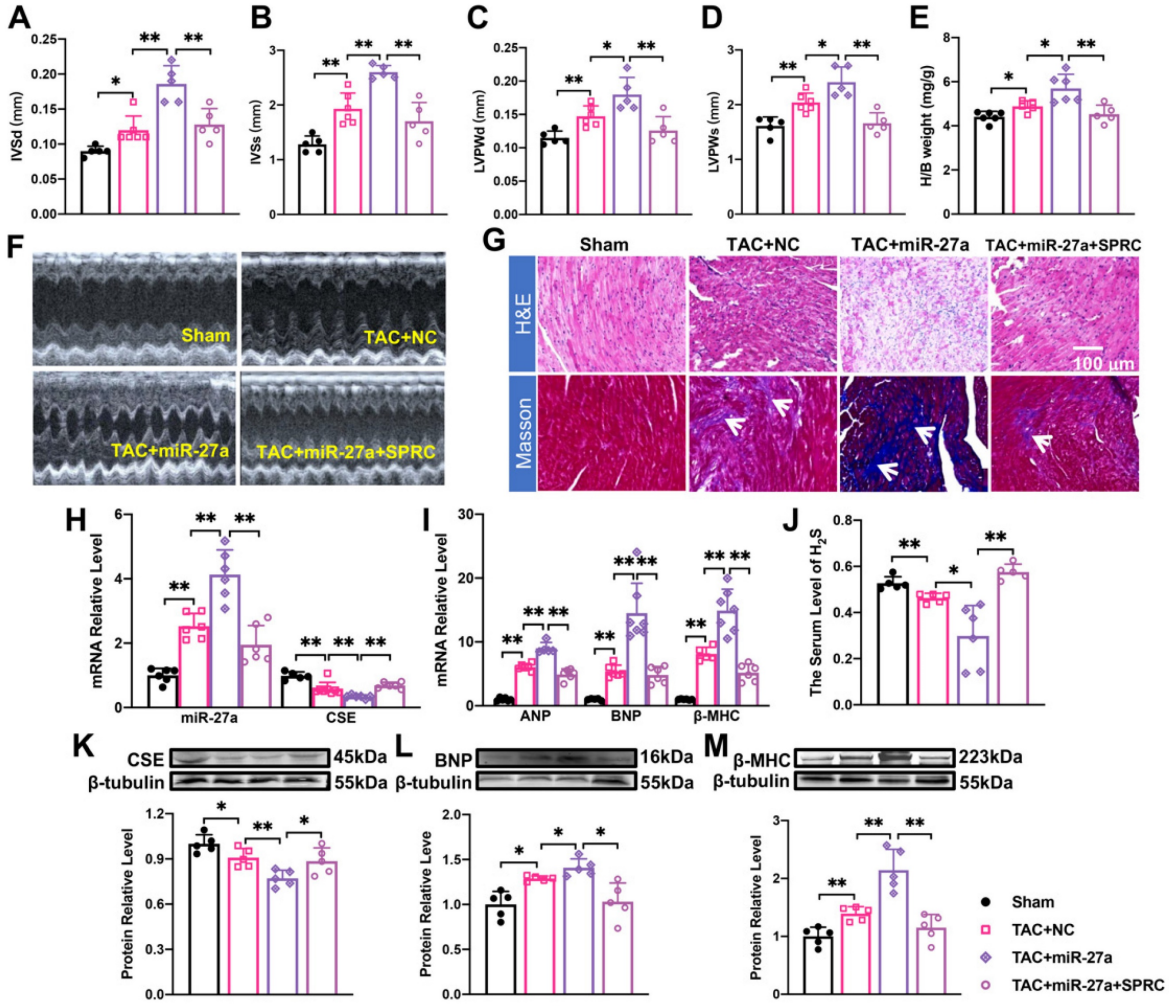
** The effect of miR-27a on myocardial hypertrophy depends on the regulation of CSE/H_2_S. (A-E).** rAAV9-miR-27a overexpressing adenovirus was injected into C57BL/6 mice at three weeks before TAC, and SPRC was intraperitoneal injected daily for eight days before surgery. Statistical results of interventricular septal thickness in diastole (IVSd, mm) and systole (IVSs, mm), left ventricular posterior wall at end-diastole (LVPWd, mm) **(C)** and end-systole (LVPWs, mm), and Heart/body weight (mg/g) in Sham, TAC + NC, TAC+ rAAV9-miR-27a (miR-27a), TAC+ miR-27a + SPRC groups (*n* = 5 mice). **(F).** Mouse echocardiogram. **(G).** Masson and HE staining were used to detect transverse sections and myocardial fibrosis in four groups. HE ×400, Masson ×400. Scale bar represents 100 μm. (*n* = 3 mice). Relative mRNA and protein expression of CSE and miR-27a **(H, K)** and hypertrophic marker factors **(I, L, M)** in the C57BL/6 was measured from Sham, TAC + NC, TAC + miR-27a, and TAC + miR-27a + SPRC by qRT-PCR and Western blot (*n* = 5-7 mice). β-tubulin served as an internal control. **(J).** Changes in the expression of H_2_S in serum (*n* = 5 group). Figure legend from **(M)** applies to all other bar graphs. Averaged data are presented as the mean ± SD; *P < 0.05, **P < 0.01. Of note, NC negative control, TAC transverse aortic constriction, SPRC S-propargyl-cysteine.

Considering that miR-27a expression was significantly increased in the cardiomyocyte hypertrophy model (Figure 1D), we hypothesized that elevated levels of miR-27a may exacerbate cardiac dysfunction. Quantification analysis confirmed that the typical echocardiographic parameters (Figure 5A-D), as well as the heart-body weight ratio (Figure 5E), induced by TAC, were more pronounced in the rAAV9-miR-27a group. Additionally, echocardiography (Figure 5F) and histological staining using Masson and HE showed more prominent abnormalities, such as increased cross-sectional area of the heart and profibrotic effects (as indicated in the figure), in the rAAV9-miR-27a group compared to the TAC group (Figure 5G). These changes were consistent with the alterations in the expression of mRNA (Figure 5I) and protein (Figure 5L, M) levels of hypertrophy markers. Notably, levels of CSE and H_2_S were downregulated in the rAAV9-miR-27a group compared to the TAC group (Figure 5H, J, K). These findings suggested that miR-27a overexpression exerted a pro-hypertrophic effect in TAC-induced cardiac hypertrophy.

To investigate the potential involvement of the CSE-mediated mechanism in the effect of miR-27a on cardiac hypertrophy, we administered S-propargyl-cysteine (SPRC), a novel CSE modulator known to enhance myocardial ischemia protection through increased H_2_S expression, through intraperitoneal injection. To further explore the effect and mechanism of AAV9-miR-27a on cardiac hypertrophy, we combined the TAC model with SPRC treatment. The results demonstrated that SPRC administration attenuated cardiac damage, as evidenced by reduced front and rear wall thickness of the heart (Figure 5A-D, F), decreased heart-body weight ratio (Figure 5E), and diminished myocardial fibrosis (Figure 5G). These changes were accompanied by a decrease in the expression of hypertrophy marker genes (Figure 5I, L, M) compared to the AAV9-miR-27a group. Additionally, the expression of miR-27a was downregulated, while the expression of CSE and H_2_S showed an opposite trend (Figure 5H, J, K). Overall, our data indicated that miR-27a overexpression could negatively regulate H_2_S production, thereby exacerbating cardiac dysfunction.

### The effect of Liraglutide/Dulaglutide on the expression of CSE and miR-27a

**Figure 6:**
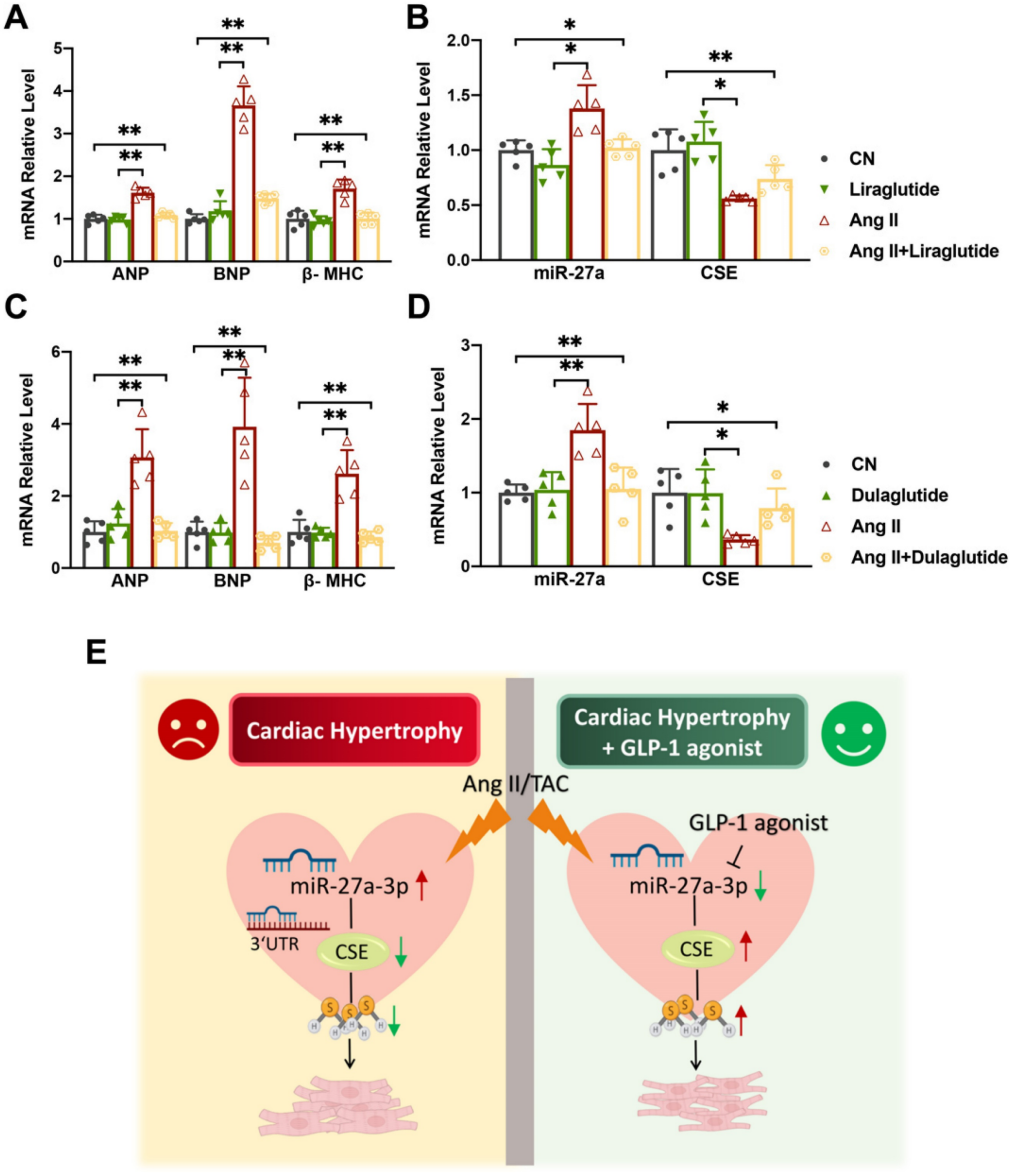
** Liraglutide/Dulaglutide protect cardiac hypertrophy by regulating CSE and miR-27a.** NMVCs were exposed to Ang II for 48 hours and Liraglutide or Dulaglutide for another 24 hours. qRT-PCR analysis of hypertrophic markers (**A, C**) and CSE/miR-27a (**B, D**) from the heart of the different groups (*n* = 5 group). Figure legend from (**B**) also applies to (**A**), figure legend from (**D**) also applies to (**C**). (**E**) Mechanism diagram of miR-27a-3p regulation on myocardial hypertrophy. Averaged data are presented as the mean ± SD; *P < 0.05, **P < 0.01. Of note, CN control, Ang II angiotensin II.

Liraglutide and dulaglutide, known as GLP-1 receptor agonists, are currently used to treat type-II diabetes and have been shown to protect against cardiac disease induced by diabetes mellitus 27, 28. Based on previous studies, we hypothesized that Liraglutide and Dulaglutide might exhibit cardioprotective effects against myocardial hypertrophy through the miR-27a/CSE pathway. To explore this, we exposed cardiomyocytes to Ang II for 48 h, followed by treatment with either Liraglutide (100 nmol/L) or Dulaglutide (100 nmol/L) for an additional 24 h. qRT-PCR and Western blot analyses revealed that both Liraglutide and Dulaglutide did not significantly affect normal cardiomyocytes, but down-regulated the expression of hypertrophy markers in model cells (Figure 6A, C). Furthermore, both treatments reduced miR-27a expression and enhanced CSE expression at both mRNA and protein levels compared to the model group (Figure 6B, D). These findings confirm that Liraglutide and Dulaglutide attenuated the progression of cardiac hypertrophy induced by TAC, which was closely associated with miR-27a and CSE. Our results provide initial support for our hypothesis, and further experiments will be conducted to confirm our conjecture.

## Discussion

Herein, we've made a groundbreaking discovery by uncovering the novel negative regulatory connection between miR-27 and CSE/H_2_S, linking it to the development of myocardial hypertrophy. This finding carries immense importance in terms of advancing future research and exploring potential treatment avenues for cardiac diseases. Research has shown that deficiency of miR-27a significantly mitigates hypertrophic responses by elevating CSE/H_2_S levels (Figure 3A-L). However, this effect is counteracted after silencing the endogenous CSE gene *in vitro*. Conversely, overexpression of miR-27a exacerbates hypertrophic responses, which can be reversed after supplementing endogenous CSE (Figure 5A-M). Additionally, we observed that liraglutide and dulaglutide (GLP-1 agonists) have an anti-hypertrophic effect. Furthermore, these medications have demonstrated the ability to reduce miR-27a levels while simultaneously increasing CSE/H_2_S levels. (Figure 6E). These findings may present new therapeutic targets for the mechanism of myocardial hypertrophy and open up new perspectives on the prevention and effective clinical treatment of heart failure.

MiR-27a and miR-27b are two subtypes from the miR-27 family that differ by only one base. They form clusters with different miRNAs and have been shown to play essential roles in various pathological conditions. For instance, miR-27a has a protective effect on lung injury by inhibiting inflammation and apoptosis, making it a potential diagnostic biomarker for acute pulmonary embolism 29, 30. Moreover, the miR-27 family is involved in systemic metabolism, participating in esterification, hydrolysis, efflux, and influx of cellular cholesterol. It also synergistically regulates adipogenesis with peroxisome proliferator activated receptor gamma (PPARG) and secretory carrier membrane protein 3 (SCAMP3), making it a potential therapeutic target in obesity-related diseases 31, 32. Furthermore, the miR-27 family is implicated in the regulation of endothelial cells, angiogenesis, and the treatment of gastric cancer 10, 33. Additionally, accumulating evidence suggests that the miR-27 family plays a significant role in the pathogenesis of cardiovascular diseases. Studies have shown that miR-27a, secreted by extracellular vesicles, aggravates cardiac hypertrophy 34. Conversely, suppression of miR-27b may decrease cardiac fibrosis and aggravate heart damage. These findings suggest that the miR-27 family is not a novel target in the field of heart disease 35, 36. However, it is the first time that the regulatory mechanism of the miR-27 family with H_2_S in relation to its impact on myocardial hypertrophy has been proposed.

Hydrogen sulfide (H_2_S) is a toxic gas that was first discovered in 1777. Our previous studies have shown that H_2_S regulates blood pressure by increasing the expression of ATP-sensitive potassium (KATP) in the baroreflex afferent pathway 37. H_2_S has also been found to have a protective effect on heart disease, as endogenous H_2_S can cross-talk with miRNA to regulate cellular processes such as fibrosis and apoptosis. This regulation alleviates pathological features such as myocardial hypertrophy and atherosclerosis. For instance, H_2_S inhibits the inactivation of Ca^2+^/calcineurin/NFATc4 signaling pathway by regulating miR-133 38, 39. Additionally, H_2_S is closely associated with miRNA. Studies have found that miR-216a and miR-21 regulate CSE reversely through multiple pathways, thus affecting systemic metabolism and cardiac changes 40, 41. CSE, as the key regulatory enzyme of H_2_S in the cardiovascular system, controls the expression of endogenous H_2_S in the heart. Our study demonstrates that miR-27a and miR-27b exert a negative regulatory effect on cystathionine gamma-lyase (CSE) by targeting the shared binding site in the 3' untranslated region (3'UTR) of the CSE gene. This contributes to the amplification of the hypertrophic response (Figure 2B, D, E). Therefore, we only selected miR-27a for subsequent verification experiments in our study. It is worth noting that the expression level of miR-27a in the heart significantly decreased after injecting SPRC in TAC mice with rAAV9-miR-27a injection. We propose two hypotheses: First, the reversal of myocardial hypertrophy leads to a decrease in miR-27a levels in mice. Second, the expression of endogenous CSE up-regulates feedback-negative regulation to the expression of miR-27a. The specific mechanism requires further verification.

Current drugs used for the clinical treatment of inflammation, aging, and associated pharmaceutical agents with cardiac hypertrophy, such as β-adrenergic receptor blockers and angiotensin II type-I receptor antagonists, have limitations 42. Therefore, it is necessary to study new antihypertrophic drugs. Glucagon-like peptide-1 (GLP-1) is an insulin-stimulating hormone secreted by L cells during food digestion in the small intestine. It promotes insulin secretion and maintains glucose homeostasis. GLP-1 receptors are widely distributed in various organs, including the pancreas, heart, lung, and skin. Liraglutide and dulaglutide are long-acting GLP-1 analogues used in the treatment of type-II diabetes. Dulaglutide has higher compliance and durability compared to liraglutide 43-45. Apart from type-II diabetes, liraglutide and dulaglutide have beneficial effects in obesity, cardiovascular diseases, and neurological diseases. The pharmacological effects and clinical applications of these drugs continue to expand 27, 28, 46-48. Cardiovascular disease is a major complication in diabetic patients, and Liraglutide was first found to reduce the rate of non-fatal myocardial infarction and stroke in type-II diabetes in 2016 23. Additionally, Liraglutide is essential in preventing cardiac dysfunction and protecting fibroblasts in diabetic rats by activating the PPARα pathway, AMPK-SIRT1 pathway, and DAG-PKC-NAD (P) H pathway to reduce oxidative stress and apoptosis 49, 50. Moreover, Liraglutide has shown a protective effect on cardiac function in non-diabetic patients, improving myocardial hypertrophy, apoptosis, interstitial myocardial fibrosis, inflammation, and oxidative stress 51. Furthermore, weekly subcutaneous injection of dulaglutide reduced the burden of cardiovascular risk factors or fatal events in patients with type-II diabetes 46. However, the effect of dulaglutide on myocardial hypertrophy has not been extensively studied. Our study demonstrates that both liraglutide and dulaglutide have the potential to mitigate the pathophysiological progression of myocardial hypertrophy. Although our *in vitro* qRT-PCR experiments confirmed that GLP-1 receptor agonists attenuate myocardial hypertrophy, which correlates with downregulation of miR-27a and upregulation of CSE/H_2_S, this does not establish a direct causal regulatory relationship. Additionally, the potential effects of blood glucose modulation by these hypoglycemic agents on myocardial tissue were not evaluated, which represents a significant oversight given the metabolic implications of glucose levels in cardiac function. Future studies are necessary to investigate these dynamics more thoroughly and to elucidate the regulatory interplay between miR-27a, CSE/H_2_S, and myocardial hypertrophy, especially in the context of fluctuating blood glucose levels. These findings provide a new theoretical basis and potential therapeutic target for using GLP-1 in the treatment of pathological cardiac hypertrophy.

In summary, our findings confirm that CSE is an effective target of miR-27a and plays a key role in the negative regulation of myocardial hypertrophy by miR-27a. Furthermore, liraglutide and dulaglutide contribute to the protection against myocardial hypertrophy through the regulation of the miR-27-CSE axis. These drugs may represent potential strategies for anti-hypertrophic therapy.

## Supplementary Material

Supplementary figure.

## Figures and Tables

**Table 1:** The list of all qRT-PCR primers used in this experiment.

Gene List	Primers	Sequences
miR-27a	Sense primer	5'-GCGCGTTCACAGTGGCTAAG-3'
	Antisense primer	5'-AGTGCAGGGTCCGAGGTATT-3'
U6	Sense primer	5'-GCTTCGGCAGCACATATACTAAAAT-3'
	Antisense primer	5'-CGCTTCACGAATTTGCGTGTCAT-3'
ANP	Sense primer	5'-TCTTCCTCGTCTTGGCCTTT-3'
	Antisense primer	5'-CCAGGTGGTCTAGCAGGTTC-3'
BNP	Sense primer	5'-TGGGAGGTCACTCCTATCCT-3'
	Antisense primer	5'-GGCCATTTCCTCCGACTTT-3'
β-MHC	Sense primer	5'-CGGACCTTGGAAGACCAGAT-3'
	Antisense primer	5'-GACAGCTCCCCATTCTCTGT-3'
CSE	Sense primer	5'-CTTGCTGCCACCATTACG-3'
	Antisense primer	5'-TTCAGATGCCACCCTCCT-3'
GAPDH	Sense primer	5'-GAACATCATCCCTGCATCCA-3'
	Antisense primer	5'-CCAGTGAGCTTCCCGTTCA-3'

## Abbreviations

AAV: Adeno-associated virus
Ang II: Angiotensin II
ANP: Atrial natriuretic peptide
BNP: Brain natriuretic peptide
β-MHC: Bata-myosin heavy chain
CSE: Cystathionine-γ-lyase
GLP-1: Glucagon-like peptide-1
HW/BW: Heart body weight ratio
H2S: Hydrogen sulfide
IVSd (s): Interventricular septal thickness in diastole (systole)
LVPWd (s): Left ventricular posterior wall at end-diastole (end-systole)
NC: Negative Control
SPRC: S-propargyl-cysteine
TAC: Transverse aortic constriction
