# Supplementary material for: The crucial relationship between miRNA-27 and CSE/H2S, and the mechanism of action of GLP-1 in myocardial hypertrophy
Source: Int J Med Sci. 2024 Mar 31;21(5):965–77. doi: 10.7150/ijms.93720 (PMC11008482; doi:10.7150/ijms.93720)

Online only Supplementary Figure

The crucial relationship between miRNA-27 and CSE/H_2S, and the mechanism of action of GLP-1 in myocardial hypertrophy

Shan Gao[1], Ying Li[1], Mei-ming Liu[1], Xue Xiong[1], Chang-peng Cui[1], Qing-ji Huo[1], Ke-xin Li[1], Xun Sun[1], Rong Zhang[1], Di Wu[1, 3]*, Bai-yan Li[1,2] *

[1] State Key Laboratory of Frigid Zone Cardiovascular Diseases (SKLFZCD), Department of Pharmacology (State Key Laboratory-Province Key Laboratories of Biomedicine-Pharmaceutics of China, Key Laboratory of Cardiovascular Research, Ministry of Education), College of Pharmacy, Harbin Medical University, Harbin 150081, China.

[2]Research Unit of Noninfectious Chronic Diseases in Frigid Zone (2019RU070), Chinese Academy of Medical Sciences, Harbin 150081, China.

[3]Department of Pharmacy, the 2nd Affiliated Hospital of Dalian Medical University, Dalian 116023, China

Supplemental Figure

S1. Validation of myocardial hypertrophy models *in vitro* and *in vivo*.

(A-E). Statistical results of interventricular septal thickness in diastole (IVSd, mm) and systole (IVSs, mm) left ventricular posterior wall at end-diastole (LVPWd, mm) and end-systole (LVPWs, mm), and heart/body weight (mg/g) in Sham and TAC group (*n* = 6-10 mice). Changes of mRNA **(F)** and protein **(G, H)** levels of cardiac hypertrophy markers, including ANP, BNP, β-MHC, in TAC mice compared with Sham group (*n* = 5 mice). β-tubulin served as an internal control (*n* = 5-6 mice or group). qRT-PCR and Western blot were used to detect the changes of mRNA **(I)** and protein **(J-L)** of hypertrophic

markers in neonatal mouse ventricular cardiomyocytes (NMVCs) after 48 hours of Ang II treatment (n = 5 group). Averaged data are presented as the mean ± SD; *$P < 0.05$, **$P < 0.01$.

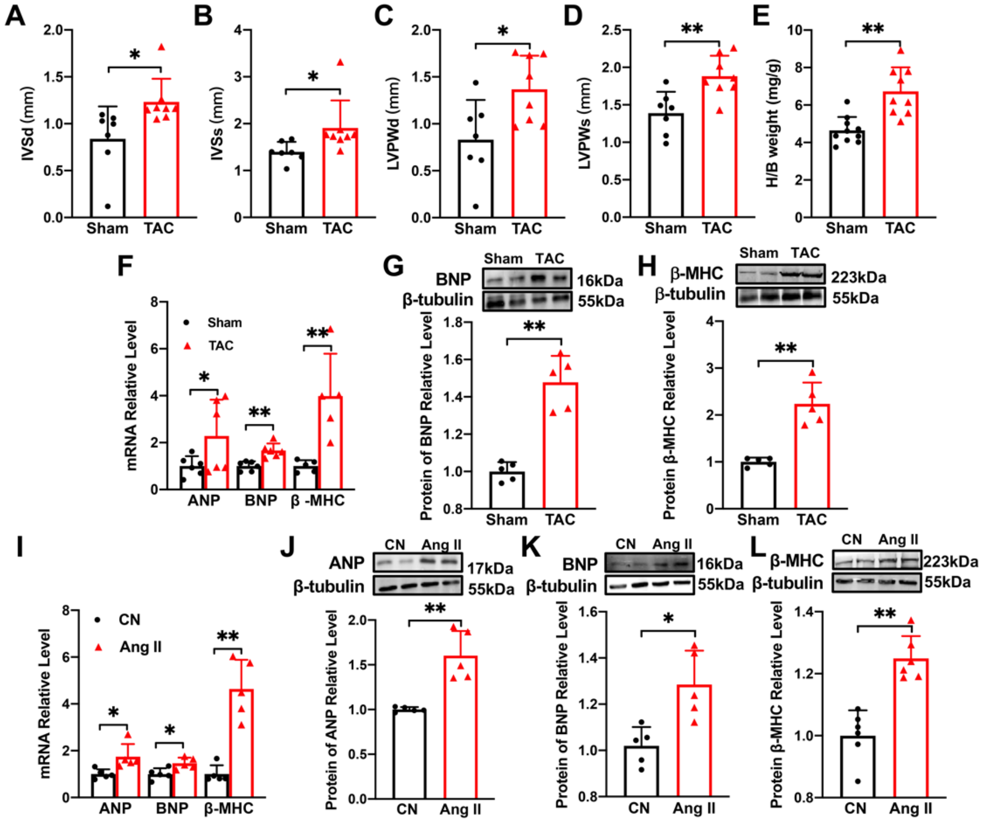

Supplement: Supplementary file 1 — Supplementary figure. [file ijmsv21p0965s1.pdf]
